# Trends in Miscarriages and Stillbirths in Panama From 2002 to 2024

**DOI:** 10.1155/ogi/2667541

**Published:** 2026-09-15

**Authors:** Yovani Chavez-Rodriguez, Tania T. Herrera R., Jessica Bahorski, Eugenia Flores Millender

**Affiliations:** ^1^ Center for Medical Research, Pacífica Salud, Hospital Punta Pacífica, Panama City, Panama; ^2^ Sistema Nacional de Investigación (SNI-AIP), SENACYT, Panama City, Panama, sni.senacyt.gob.pa; ^3^ College of Nursing, Florida State University, Tallahassee, Florida, USA, fsu.edu; ^4^ Center of Population Sciences for Health Equity, Florida State University, Tallahassee, Florida, USA, fsu.edu

## Abstract

**Introduction:**

Pregnancy loss, including miscarriage and stillbirth, remains a significant public health concern, particularly in low‐ and middle‐income countries. Reliable national‐level data are essential to monitor trends and guide interventions. This study aims to describe temporal trends in pregnancy losses in Panama over the past 2 decades, using national vital statistics.

**Methods:**

We conducted a population‐based, retrospective analysis of pregnancy outcomes in the Republic of Panama between 2002 and 2024. Data were obtained from the Panama National Institute of Statistics and Census, which compiles vital statistics from health facilities nationwide. The dataset included all registered pregnancies resulting in live birth, miscarriage, or stillbirths. Causes for miscarriage and stillbirth were classified using ICD‐10 coding categories. All analyses were conducted using IBM SPSS Statistics Version 29 and Joinpoint Regression Program version 6.0.

**Results:**

This study registered 190,764 miscarriages, 15,904 stillbirths, and 1,582,352 live births, which represented an average raw pregnancy loss rate of 115.6 (95% CI 110.7–120.4) per 1000 total registered pregnancies in Panama from 2002 to 2024. While the average miscarriage rate was 106.6 (95% CI 101.9–111.4) per 1000 total registered pregnancies, the average stillbirth rate was 10.0 (95% CI 9.6–10.4) per 1000 total births. In general, 11.6% of all registered pregnancies in Panama ended in pregnancy loss. Rates of pregnancy loss were highest among women aged ≥ 40 years and single women. Joinpoint regression analysis identified significant declines in miscarriage rates (2008–2021) and stillbirth rates (2002–2017), but neither was sustained through 2024. All miscarriage cases were coded exclusively as P01, reflecting a complete absence of diagnostic diversity. Among stillbirths, P95 (unspecified fetal death; 53.4%), P02 (placenta, cord, and membranes; 21%), and P20 (intrauterine hypoxia; 7.4%) together accounted for over 80% of cases.

**Conclusion:**

Pregnancy loss rates in Panama have remained stagnant over the past 2 decades, despite the establishment of national surveillance policies and reporting mechanisms. Miscarriage and stillbirth trends showed limited and unsustainable improvements. The overwhelming reliance on a narrow set of ICD‐10 codes highlights systemic weaknesses in perinatal cause of death classification that limits opportunities for prevention. Strengthening perinatal pathology capacity, coder training and equitable care access must be strengthened.

**Summary**
• Synopsis
◦ Study question: What are the long‐term trends in miscarriage and stillbirth rates and causes of death in Panama over the last 2 decades?
• What’s already known?
◦ Global declines in stillbirth have been observed, but progress is uneven. Many low‐ and middle‐income countries lack reliable data on pregnancy loss, and ICD‐10 coding often obscures underlying causes.
• What this study adds?
◦ Using 23 years of national data, this study shows that although Panama seems to be on track to fulfill strategic objectives, the nation’s pregnancy loss rates in Panama have not been constant. Reliance on limited ICD‐10 categories constrains interpretation. These findings highlight the urgent need to strengthen perinatal surveillance, diagnostic practices, and maternal health policy to reduce preventable losses and align Panama with global pregnancy loss reduction targets.

## 1. Introduction

Pregnancy loss, encompassing miscarriage and stillbirth, is a key indicator of maternal and perinatal health, reflecting both the quality of obstetric care and broader determinants of women’s health [1]. Miscarriage, defined as loss before 20 weeks’ gestation, and stillbirth, occurring at or after 20 weeks, together account for a substantial proportion of adverse pregnancy outcomes worldwide [2]. Despite advances in prenatal diagnosis and care, many cases remain unexplained, often attributed to genetic, maternal, behavioral, or environmental risk factors [3–13]. The psychosocial burden of pregnancy loss is also profound, contributing to depression, anxiety, posttraumatic stress, and long‐term cardiovascular morbidity in affected women [14–18].

Global initiatives underscore the urgency of addressing pregnancy loss. The Sustainable Development Goals (SDGs) call for the end of preventable maternal and child deaths, with specific targets for improving antenatal care and strengthening health systems. The Every Newborn Action Plan and the World Health Organization (WHO) 2030 aim to reduce stillbirth rates to ≤ 12 per 1000 total births, highlighting the importance of robust surveillance and responsive policies to achieve equity in outcomes [19, 20].

Despite these commitments, progress has been uneven. Between 2000 and 2015, global stillbirth rates declined by 19%, but most improvements occurred in high‐income countries [21]. In low‐ and middle‐income countries (LMICs), stillbirth rates remain disproportionately high, with persistent regional and socioeconomic disparities.

Latin America and the Caribbean exemplify this uneven progress. While Brazil has documented declines in fetal deaths, entrenched regional disparities remain, and Caribbean nations struggle with fragmented surveillance and underreporting [22, 23]. Few studies have assessed long‐term trends in pregnancy loss in Central America, leaving critical evidence gaps for the region.

Panama, an upper‐middle‐income country in Latin America with advanced urban health infrastructure but also underserved rural and Indigenous populations, represents an important case study. It offers insight into how national policies translate or fail to translate into population‐level outcomes in LMICs, where equity gaps and health system capacity remain major challenges.

Panama has enacted several policy measures to strengthen maternal and perinatal surveillance. Resolution 93 (2001) established a National, Interinstitutional, and Intersectoral Commission on Maternal and Perinatal Mortality to coordinate monitoring of pregnancy outcomes [24, 25]. Executive Decree 1617 (2014) mandated formal notification of pregnancy loss using International Classification of Diseases, 10th Revision (ICD‐10 coding) to improve national surveillance [26]. More recently, Executive Decree 932 (2021) adopted ICD‐11 to modernize classification and improve accuracy [27]. While these policies reflect national commitment, concerns remain regarding the reliability of ICD‐10 coding in perinatal settings, where nonspecific categories are often overused [28–30].

This study builds directly on global commitments such as the Every Newborn Action Plan and WHO 2030 stillbirth reduction targets, as well as regional maternal health equity priorities [20, 21, 28]. By analyzing 23 years of national surveillance data, we evaluate whether Panama’s policy initiatives—specifically Resolution 93 (2001) and Executive Decree 1617 (2014)—have translated into measurable reductions in pregnancy loss [24, 26]. This work addresses critical evidence gaps in Latin America, where reliable national data remain limited and coding practices often hinder causal interpretation [22, 29, 30].

Our primary objective was to evaluate trends in miscarriage and stillbirth rates, stratified by gestational age and cause of death. Our secondary objective was to assess variations in maternal demographics, including age, marital status, and province of residence. Despite more than 2 decades of surveillance efforts, it remains unknown whether pregnancy loss rates in Panama have improved [24, 26, 28].

## 2. Methodology

### 2.1. Study Design and Data Source

We conducted a population‐based, retrospective analysis of pregnancy outcomes in the Republic of Panama between 2002 and 2024. Data were obtained from the Panama National Institute of Statistics and Census (INEC in Spanish), which compiles vital statistics from health facilities nationwide [28]. The dataset included all registered pregnancies resulting in live births, miscarriages, and stillbirths.

### 2.2. Case Definitions

All miscarriage and stillbirth data were extracted from the INEC registry. Pregnancy losses (miscarriage and stillbirth) were classified using ICD‐10 codes [31, 32]. Miscarriage, also known as spontaneous abortion or intrauterine pregnancy loss, is defined as a pregnancy loss before 20 weeks’ gestation, while stillbirth is the death of a fetus at or after 20 weeks’ gestation [33]. Stillbirths were subdivided following United States Centers for Disease Control and Prevention (CDC) definitions: early stillbirth (20–27 weeks), late stillbirth (28–35 weeks), and term stillbirth (≥ 36 weeks) [34]. Causes of death for miscarriage and stillbirth were classified using ICD‐10 coding categories, which included P00 (fetus/newborn affected by maternal conditions), P01 (maternal complications of pregnancy), P02 (placenta, cord, and membranes), P03 (labor and delivery complications), P07 (short gestation and low birthweight), P20 (intrauterine hypoxia), P95 (unspecified fetal death), Q89 (other congenital malformations), and other specified causes [32, 33].

### 2.3. Covariates

Maternal demographic characteristics included age: < 15, 15–19, 20–29, 30–39, 40–49, and ≥ 50; marital status: single, cohabiting, married, and separated/widowed; and province of occurrence: Bocas del Toro, Coclé, Colón, Chiriquí, Darién, Herrera, Los Santos, Panamá/Panamá Oeste, Veraguas, and Indigenous Comarcas. Provinces were coded as urban, rural, extremely rural, and indigenous comarcas.

### 2.4. Statistical Analysis

Raw pregnancy loss, also known as pregnancy waste ratio, was calculated as the sum of miscarriages and stillbirths divided by total registered pregnancies and then multiplied by 1000. Specifically, the miscarriage rate was calculated as the total number of miscarriages divided by the total number of registered pregnancies during that period multiplied by 1000. Stillbirths were divided into early, late, and term; then, the rate per 1000 total births (live births + stillbirths) for each category was calculated using the same technique [28]. Results are presented as rates with 95% confidence intervals (CI). CI was calculated using the point estimate ± margin of error method [31, 32, 35].

(1) Raw pregnancy loss rate=Number of pregnancy losses miscarriages+stillbirthsTotal number of registered pregnancies live births+miscarriages+stillbirths×1000,

(2) Miscarriage rate=Number of miscarriagesTotal registered pregnancies live births+miscarriages+stillbirths×1000,

(3) Stillbirth rate=Number of stillbirthsTotal births live births+stillbirths×1000.

### 2.5. Missing Data

Unspecified maternal age was the most frequent missing variable but represented a small fraction of records (0.8%) [31]. There was no missing data for marital status or province of residence. These cases were retained in analyses to avoid underestimation of losses, consistent with best practices for handling incomplete demographic data [36].

Sensitivity analyses comparing crude estimates with age‐stratified models confirmed consistency of patterns, suggesting minimal bias from missing data [36]. Reporting of missing data handling follows STROBE recommendations for observational studies [37].

### 2.6. Justification of Analytic Approach

Analyzing trends among miscarriages and stillbirths allows a more profound understanding of cause of death, which may elucidate present problems and possible courses of action. By using Joinpoint trend regression analysis, can identify statistically significant changes in trends and it was thus used in these analyses [38].

### 2.7. Software

All analyses were conducted using IBM SPSS Statistics Version 29 (Armonk, NY, USA) [39] and Joinpoint Regression Program Version 6.0 [38].

### 2.8. Ethical Considerations

This study analyzed publicly available, de‐identified aggregate data. No individual‐level information was included, and confidentiality was not compromised. In accordance with national and international standards, ethical approval was not required.

## 3. Results

This study registered 190,764 miscarriages, 15,904 stillbirths, and 1,582,352 live births, which represented an average raw pregnancy loss rate of 115.6 (95% CI 110.7–120.4) per 1000 total registered pregnancies in Panama from 2002 to 2024. While the average miscarriage rate was 106.6 (95% CI 101.9–111.4) per 1000 total registered pregnancies, the average stillbirth rate was 10.0 (95% CI 9.6–10.4) per 1000 total births. In general, 11.6% of all registered pregnancies in Panama ended in pregnancy loss (Table 1).

**TABLE 1:** Pregnancy loss rates from 2002 to 2024.

Years	Number of live birth	Raw pregnancy loss rate		Miscarriage rate		Stillbirth rate	
		Number	Rate	Number	Rate	Number	Rate
2002	61,671	8166	116.9	7481	107.1	685	11.0
2003	61,753	8651	122.9	7924	112.6	727	11.6
2004	62,743	9326	129.4	8654	120.1	672	10.6
2005	63,645	9030	124.3	8279	113.9	751	11.7
2006	65,764	9760	129.2	9048	119.8	712	10.7
2007	67,364	10,024	129.5	9302	120.2	722	10.6
2008	68,759	10,292	130.2	9496	120.1	796	11.4
2009	68,364	10,267	130.6	9583	121.9	684	9.9
2010	67,955	9900	127.2	9209	118.3	691	10.1
2011	73,292	10,078	120.9	9346	112.1	732	9.9
2012	75,486	9801	114.9	9053	106.1	748	9.8
2013	73,804	9664	115.8	8971	107.5	693	9.3
2014	75,183	9182	108.8	8536	101.2	646	8.5
2015	75,901	9474	111.0	8703	101.9	771	10.1
2016	75,184	9431	111.5	8794	103.9	637	8.4
2017	76,166	9507	111.0	8830	103.1	677	8.8
2018	76,863	9271	107.6	8573	99.5	698	9.0
2019	72,456	9024	110.8	8322	102.1	702	9.6
2020	69,945	6868	89.4	6274	81.7	594	8.4
2021	66,498	7255	98.4	6522	88.4	733	10.9
2022	63,920	7160	100.7	6514	91.6	646	10.0
2023	59,907	7173	106.9	6543	97.5	630	10.4
2024	59,729	7364	109.8	6807	101.5	557	9.2
Total	**1,582,352**	**206,668**	**AV = 115.6 (95% CI 110.7; 120.4)**	**190,764**	**AV = 106.6 (95% CI 101.9; 111.4)**	**15,904**	**AV = 10.0 (95% CI 9.6; 10.4)**

*Note:* Bold values indicate totals for 2002–2024 and the corresponding average rates.

Abbreviations: AV, average; CI, confidence interval.

Nearly half (48.9%) of mothers were aged 20–29 years. Most (75.4%) were cohabitating with a partner or married, and 68.8% were living in an urban area (Table 2). Pregnancy loss rates increased with maternal age. Women older than 40 years had the highest loss rate.

**TABLE 2:** Covariates.

Maternal age	Number of live birth y maternal age	Number of pregnancy losses	Raw pregnancy loss rate by maternal age	Average raw pregnancy loss rate per year	95% CI (LB)	95% CI (UB)	Percentage (%)
> 15 years	11672.0	1247.0	96.5	95.2	88.0	102.4	0.6
15–19 years	277943.0	28189.0	92.1	91.3	85.4	97.2	13.6
20–29 years	830880.0	101084.0	108.5	108.5	103.3	113.7	48.9
30–39 years	418212.0	62696.0	130.4	130.8	125.8	135.7	30.3
40–49 years	39348.0	11728.0	229.6	229.8	221.5	238.2	5.7
≥ 50 years	277.0	83.0	230.6	245.8	186.3	305.3	0.0
Undetermined age	4020.0	1641.0	289.9	376.1	295.7	456.5	0.8

**Marital status**	**Number of live births by marital status**	**Number of pregnancy losses by marital status**	**Raw pregnancy loss rate by marital status**	**Average raw pregnancy loss rate per year**	**95% CI (LB)**	**95% CI (UB)**	**Percentage (%)**

Single	239153.0	50381.0	174.0	176.7	160.7	192.7	24.4
Separated/Widowed	3968.0	474.0	106.7	114.9	84.2	145.6	0.2
Cohabitation	1096899.0	129987.0	105.9	106.1	102.1	110.2	62.9
Married	242332.0	25826.0	96.3	95.3	90.5	100.1	12.5

**Place of occurrence**	**Number of live birth by provinces**	**Number of pregnancy losses**	**Raw pregnancy loss rate by provinces**	**Average raw pregnancy loss rate per year**	**95% CI (LB)**	**95% CI (UB)**	**Percentage (%)**

Panama/P. Oeste (Urban)	763850.0	123811.0	139.5	139.3	132.2	146.4	59.9
Colon (Urban)	118222.0	18406.0	134.7	135.3	129.4	141.3	8.9
Herrera (Rural)	36951.0	5191.0	123.2	123.9	118.5	129.3	2.5
Los Santos (Rural)	24958.0	3492.0	122.7	123.0	112.2	133.7	1.7
Chiriqui (Mixed)	185921.0	22813.0	109.3	109.2	105.0	113.3	11.0
Veraguas (Rural)	93573.0	9541.0	92.5	92.6	89.5	95.7	4.6
Cocle (Rural)	94582.0	9151.0	88.2	87.7	81.9	93.4	4.4
Bocas del Toro (Rural)	92274.0	7869.0	78.6	79.3	74.3	84.2	3.8
Darien (Rural)	23378.0	1467.0	59.0	59.7	49.9	69.5	0.7
Indigenous regions (Rural)	148643.0	4927.0	32.1	32.6	30.5	34.7	2.4

### 3.1. Maternal Demographics

Marital status distributions differed significantly, with the highest average raw pregnancy loss rates found in single women: 176.7 (95% CI 160.7–192.7) per 1000 total registered pregnancies. Separated/widowed women had a rate of 114.9 (95% CI 84.2–145.6) per 1000 total registered pregnancies. Cohabitating women registered a rate of 106.1 (95% CI 102.1–110.2) per 1000 total registered pregnancies. The lowest average raw pregnancy loss rate was found among married women, with a rate of 95.3 (95% CI 90.5–100.1) per 1000 total registered pregnancies (Table 3).

**TABLE 3:** Causes of death by ICD‐10.

Pregnancy loss period	ICD‐10 code	Number of miscarriages							Rate per 1000 total registered pregnancies	Average rate per 1000 total registered pregnancies per year	95% CI (LB) rate	95% CI (UB) rate	Percentage (%)
Miscarriage (< 20 weeks)	P01	190,764							106.6	106.6	101.9	111.4	100.0
Total		190,764							106.6	106.6	101.9	111.4	100.0

**Pregnancy loss period**	**ICD-10 code**	**Number of early stillbirth**	**Rate of early stillbirth**	**Number of late stillbirths**	**Rate of late stillbirth**	**Number of term stillbirths**	**Rate of term stillbirth**	**Total number of stillbirths**	**Rate per 1000 total births**	**Average rate per 1000 total births per year**	**95% CI (LB) Rate**	**95% CI (UB) Rate**	**Percentage (%)**

Stillbirth (≥ 20 weeks)	P00	126	0.1	201	0.1	200	0.1	527	0.3	0.3	0.3	0.4	3.3
	P01	282	0.2	88	0.1	134	0.1	504	0.3	0.3	0.2	0.4	3.2
	P02	775	0.5	1111	0.7	1455	0.9	3341	2.1	2.1	1.9	2.3	21.0
	P07	468	0.3	73	0.0	34	0.0	575	0.4	0.4	0.3	0.4	3.6
	P20	334	0.2	338	0.2	502	0.3	1174	0.7	0.7	0.6	0.9	7.4
	P95	4396	2.8	1700	1.1	2393	1.5	8489	5.3	5.4	4.9	5.9	53.4
	Q89	157	0.1	124	0.1	96	0.1	377	0.2	0.2	0.2	0.3	2.4
	Others	226	0.1	259	0.2	432	0.3	917	0.6	0.6	0.5	0.7	5.8
Total		6764	4.2	3894	2.4	5246	3.3	15,904	10.0	10.0	9.6	10.4	100.0

Geographic disparities were notable: urban provinces accounted for the highest average pregnancy loss rates. For example, while Panama/Panama Oeste registered an average raw pregnancy loss rate of 139.3 (95% CI 132.2–146.4), Colon’s average rate was 135.3 (95% CI 129.4–141.3). Indigenous Comarcas and extremely rural areas consistently reported the lowest pregnancy loss rates. For instance, Darien registered an average raw pregnancy loss rate of 49.9 (95% CI 49.9–69.5), while Indigenous regions registered a rate of 32.6 (95% CI 30.5–34.7). However, these lower rates in rural and Indigenous regions may reflect underreporting and surveillance limitations rather than reduced risk exposures (Table 3).

### 3.2. Joinpoint Regression Trend Analysis

#### 3.2.1. Miscarriage Rate Trends

**FIGURE 1:**
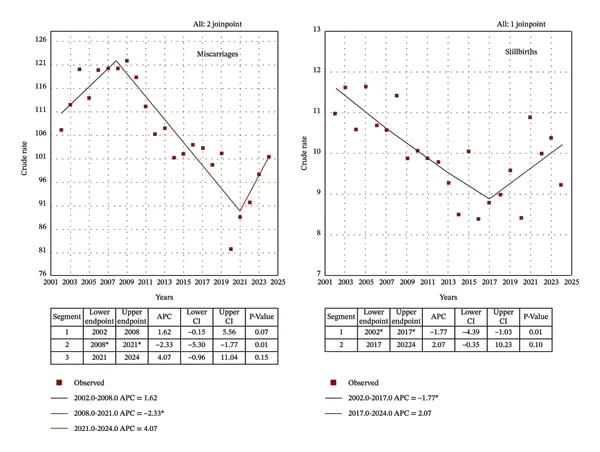
Miscarriage and stillbirth trends.

From 2002 to 2024, the miscarriage rate in Panama was 106.6 (95% CI 101.9–111.4) per 1000 total registered pregnancies. The Jointpoint regression analysis revealed three segments. The miscarriage rate trend showed an initial nonsignificant increase from 2002–2008 (APC = 1.62%; 95% CI −0.15–5.56; *p* value = 0.07), followed by a significant decline from 2008 to 2021 (APC = −2.33%; 95% CI −5.30–−1.77; *p* value = 0.01), and a nonsignificant increase from 2021 to 2024 (APC = 4.07, 95% CI −0.96–11.04; *p* value = 0.15) (Figure 1).

#### 3.2.2. Stillbirth Trends

From 2002 to 2024, the stillbirth rate was 10.0 (95% CI 9.6–10.4) per 1000 total births. The Joinpoint regression analysis revealed two segments. The miscarriage rate trend showed an initial significant decrease from 2002–2017 (APC = −1.77%; 95% CI −4.39–−1.03; *p* value = 0.01), followed by a nonsignificant increase from 2017–2024 (APC = 2.07%; 95% CI −0.36–10.23; *p* value = 0.10) (Figure 1).

#### 3.2.3. Cause‐of‐Death Coding

**FIGURE 2:**
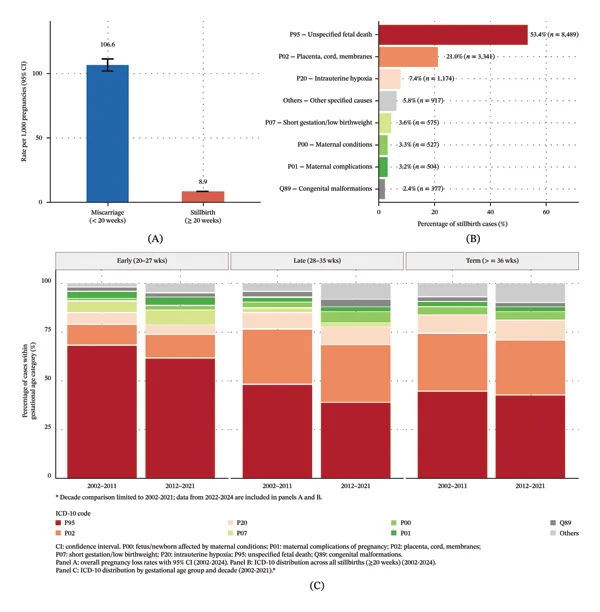
Pregnancy loss rates and ICD‐10 causes of death coding in Panama 2002–2024.

Miscarriage coding lacked diagnostic diversity: 100% of cases were assigned to P01 (fetus and newborn affected by maternal complications of pregnancy). Among stillbirths, three ICD‐10 [28, 29, 32] categories dominated: P02 (placenta, cord, membranes), P20 (intrauterine hypoxia), and P95 (unspecified fetal death), together accounting for > 80% of cases across gestational age groups and decades (Figure 2). The most common cause was unspecified fetal mortality (P95), which accounted for 53.4% of cases (*n* = 8489), followed by placental, cord, and membrane problems (P02; 21.0%, *n* = 3341) and intrauterine hypoxia (P20; 7.4%, *n* = 1174). The remaining cases included short gestation or low birthweight (P07; 3.6%), fetus/newborns affected by maternal conditions (P00; 3.3%, *n* = 527), maternal pregnancy problems (P01; 3.2%, *n* = 504), other specified causes (5.8%, *n* = 917), and congenital anomalies (Q89; 2.4%, *n* = 377) are among the remaining ICD‐10 categories.

The Joinpoint regression analysis for stillbirth trends indicated that P02 (placenta, cord, and membranes) demonstrated a significant increase from 2002 to 2010 (APC = +7.77%; 95% CI: 3.37–19.56; *p* < 0.01), followed by a significant decrease from 2010 to 2024 (APC = −3.94%; 95% CI: −7.51–−2.16; *p* < 0.01), whereas P20 (intrauterine hypoxia) exhibited a nonsignificant decreasing trend across the entire study period (APC = −3.25; 95% CI: −6.65–−0.21; *p* > 0.05) (Figure S1).

## 4. Discussion

This national analysis revealed an average raw pregnancy loss rate of 115.6 (95% CI: 110.7–120.4) per 1000 total registered pregnancies in Panama from 2002 to 2024, with a miscarriage rate of 106.6 per 1000 total registered pregnancies (95% CI 101.9–111.4) and a stillbirth rate of 10.0 (95% CI 9.6–10.4) per 1000 total births, representing 11.6% of all registered pregnancies. Although significant declines were observed—miscarriages from 2008 to 2021 and stillbirths from 2002 to 2017—these improvements were not sustained across the full study period, suggesting that existing surveillance policies have yet to translate into durable epidemiological progress [24–26].

### 4.1. Health Equity and Social Determinants

Pregnancy loss is influenced by both biological and social determinants of health [16, 17, 40]. In Panama, disparities emerged by geography and marital status. Urban provinces reported the highest number of losses, whereas Indigenous and rural provinces showed lower but persistent rates. These differences likely reflect variation in access to care, reporting accuracy, and health system capacity [22]. Indigenous and remote regions often face geographic isolation, limited infrastructure, and underreporting, which may underestimate their true burden [40–42]. By contrast, urban provinces, although better resourced, may experience both stronger surveillance and inequities in responsiveness to vulnerable women. Poverty, social exclusion, and limited antenatal care remain critical drivers of inequity [40, 43, 44].

### 4.2. International and Regional Comparisons

Panama’s stagnant rates contrast with declines observed in high‐income countries [44]. In Ireland and Finland, standardized obstetric protocols, expanded access to ultrasound, and enhanced fetal surveillance systems have contributed to measurable reductions in pregnancy loss [2, 35]. In the United States, fetal mortality rates have steadily declined, reflecting improvements in surveillance and risk screening [44]. By comparison, progress in Latin America and the Caribbean has been uneven [44, 45]. Brazil reported overall reductions in fetal death rates but persistent social and regional inequalities [45], while Caribbean nations face fragmented surveillance and underreporting. Panama’s experience mirrors these regional challenges, highlighting systemic inequities and weak data systems as barriers to progress. Strengthening national data is essential not only for domestic policy but also for alignment with global commitments such as the Every Newborn Action Plan and the WHO 2030 goal of reducing stillbirth rates to ≤ 12 per 1000 total births [20, 21].

### 4.3. ICD‐10 Coding Limitations

A major barrier identified in this study is the lack of diagnostic diversity in ICD‐10 coding. All miscarriages were classified as maternal complications of pregnancy (P01), and over 80% of stillbirths clustered into three codes: placental/membrane complications (P02), intrauterine hypoxia (P20), and unspecified fetal death (P95) [33, 44]. This lack of specificity obscures underlying causes and limits opportunities for prevention.

Among stillbirths, three codes—P02 (placenta, cord, and membranes), P20 (intrauterine hypoxia), and P95 (unspecified fetal death)—collectively accounted for over 80% of cases. In comparison, a Brazilian multicenter study from 2009 to 2018 found that 40.8% of fetal deaths were coded as P20 and P95 [46], suggesting that while diagnostic concentration is a regional pattern, Panama represents an extreme case.

Several interrelated systemic factors contribute to this problem. Panama faces a critical shortage of trained perinatal pathologists, maternal‐fetal medicine specialists, and obstetricians with advanced training in ultrasound, limiting the nation’s capability for detailed cause‐of‐death investigation [47, 48]. Underreporting in rural and Indigenous regions further skews data, rendering vulnerable populations invisible [22]. A slight misalignment between Panama’s National Guide to Epidemiology, which prioritizes P95 for pregnancy losses [48–50], and WHO standards recommending broader use of perinatal and congenital anomaly codes [41], encourages default coding. Insufficient training and resources for clinicians and coders reinforce these practices, perpetuating poor data quality [28, 29].

### 4.4. Strategies for Improvement

Addressing pregnancy loss in Panama requires a coordinated multilevel response. Mandatory stillbirth audits and structured coder training aligned with WHO standards—incorporating placental pathology, maternal interviews, and medical and image record reviews—are essential to reduce reliance on unspecified codes, supported by PAHO collaboration for cross‐country benchmarking [51–53]. Investment in perinatal pathology capacity, including autopsies, placental histology, and genetic testing, should be systematically expanded through academic partnerships, and maternal fetal medicine subspeciality residency programs should be developed [47, 48, 51, 54]. Equitable antenatal care access must be prioritized: the WHO recommends a minimum of eight antenatal contacts [55], yet Panama endorses only four, leaving many women—particularly those in Indigenous and rural communities—with insufficient monitoring to detect and manage preventable risk factors [22]. Digital surveillance platforms and mobile health tools could strengthen real‐time reporting and extend community health worker capacity in underserved regions [41, 42].

### 4.5. Strengths and Limitations

While this study used the CDC guidelines, which consider losses after 20 weeks stillbirths, its results could not be fully compared to those of other studies, which used WHO guidelines that consider losses after 28 weeks stillbirths. Although miscarriage rates used total registered pregnancies as denominators, stillbirth rates used total births, which may underestimate the risk at earlier gestational ages.

Additionally, reliance on routine ICD‐10 administrative coding introduces potential misclassification bias, and underreporting in Indigenous and rural regions may result in underestimation of the true national burden of pregnancy loss.

## 5. Conclusions

This study demonstrates that pregnancy loss rates in Panama have remained stagnant over the past 2 decades, despite the establishment of national surveillance policies and reporting mechanisms. Miscarriage and stillbirth trends showed little improvement, which was not sustained, and the overwhelming reliance on a narrow set of ICD‐10 codes highlights systemic weaknesses in perinatal classification.

These findings point to broader challenges: insufficient diagnostic capacity and misalignment between national guidelines and international standards. The persistence of high miscarriage rates and stagnant stillbirth trends underscores the urgent need for targeted action. Enhancing perinatal surveillance systems, expanding access to high‐quality antenatal care, and strengthening diagnostic investigation—including placental pathology and maternal death audits—are critical steps toward addressing preventable pregnancy losses. Digital health innovations and regional collaboration through mechanisms such as the PAHO could help overcome infrastructure and training barriers.

Beyond national importance, Panama’s experience offers lessons for other middle‐income countries struggling to translate policy into measurable reductions in pregnancy loss. By aligning with global strategies such as the WHO Every Newborn Action Plan and the SDGs, Panama could lead in advancing maternal and perinatal health equity in Latin America.

Improved coding, surveillance, and equitable maternal care are essential for Panama to meet miscarriage and stillbirth reduction targets and provide a model for similar middle‐income countries in Latin America.

## Funding

No funding was received for this manuscript.

## Conflicts of Interest

The authors declare no conflicts of interest.

## Supporting Information

Additional supporting information can be found online in the Supporting Information section.

## Supporting information


**Supporting Information 1** Figure S1. Joinpoint regression analysis of ICD‐10 codes for causes of death among stillbirths in Panama.

**Supporting Information 2** STROBE Statement—checklist of items that should be included in reports of observational studies.

## Data Availability

The data that support the findings of this study are available in INEC. Vital Statistics at https://www.inec.gob.pa. These data were derived from the following resources available in the public domain: Estadísticas Vitales, https://www.inec.gob.pa/publicaciones/Default2.aspx?ID_CATEGORIA=3%26ID_SUBCATEGORIA=6.
